# A single amino acid mutation in the mouse MEIG1 protein disrupts a cargo transport system necessary for sperm formation

**DOI:** 10.1016/j.jbc.2021.101312

**Published:** 2021-10-19

**Authors:** Wei Li, Qian Huang, Ling Zhang, Hong Liu, David Zhang, Shuo Yuan, Yitian Yap, Wei Qu, Rita Shiang, Shizheng Song, Rex A. Hess, Zhibing Zhang

**Affiliations:** 1 Department of Physiology, Wayne State University, Detroit, Michigan, USA; 2 Department of Occupational and Environmental Health, School of Public Health, Wuhan University of Science and Technology, Wuhan, Hubei, China; 3 School of Arts and Sciences, College of William and Mary, Williamsburg, Virginia, USA; 4 Department of Human and Molecular Genetics, Virginia Commonwealth University, Richmond, Virginia, USA; 5 Department of Comparative Biosciences, College of Veterinary Medicine, University of Illinois, Urbana, Illinois, USA; 6 Department of Obstetrics and Gynecology, Wayne State University, Detroit, Michigan, USA

**Keywords:** MEIG1, PACRG, SPAG16, manchette, cargo transport, single amino acid mutation, spermiogenesis, male fertility, IMT, intra-manchette transport, MEIG1, meiosis-expressed gene 1, NMR, nuclear magnetic resonance, PACRG, Parkin coregulated gene, PAM, protospacer adjacent motif, PBS, phosphate-buffered saline, SPAG16, sperm-associated antigen 16

## Abstract

Mammalian spermatogenesis is a highly coordinated process that requires cooperation between specific proteins to coordinate diverse biological functions. For example, mouse Parkin coregulated gene (PACRG) recruits meiosis-expressed gene 1 (MEIG1) to the manchette during normal spermiogenesis. Here we mutated Y68 of MEIG1 using the CRISPR/cas9 system and examined the biological and physiological consequences in mice. All homozygous mutant males examined were completely infertile, and sperm count was dramatically reduced. The few developed sperm were immotile and displayed multiple abnormalities. Histological staining showed impaired spermiogenesis in these mutant mice. Immunofluorescent staining further revealed that this mutant MEIG1 was still present in the cell body of spermatocytes, but also that more MEIG1 accumulated in the acrosome region of round spermatids. The mutant MEIG1 and a cargo protein of the MEIG1/PACRG complex, sperm-associated antigen 16L (SPAG16L), were no longer found to be present in the manchette; however, localization of the PACRG component was not changed in the mutants. These findings demonstrate that Y68 of MEIG1 is a key amino acid required for PACRG to recruit MEIG1 to the manchette to transport cargo proteins during sperm flagella formation. Given that MEIG1 and PACRG are conserved in humans, small molecules that block MEIG1/PACRG interaction are likely ideal targets for the development of male contraconception drugs.

Mouse *Meig1* was originally cloned in a screen for genes essential for meiosis (1). Multiple *Meig1* transcripts are present in different tissues encoding the same protein but differing in their 5′- or 3′-UTRs (1, 2). MEIG1 protein is found in other species, and the amino acid sequences are highly conserved (2, 3). Even though *Meig1* is expressed in multiple tissues, it is most abundantly expressed in tissues rich in ciliated cells. Therefore, it is predicted to be important for cilia formation. In mouse testis, *Meig1* message is present in germ cells and Sertoli cells (4, 5, 6, 7, 8). Global *Meig1* knockout mice showed pure male infertility due to impaired spermiogenesis, but no meiosis defect was found (2, 3). Our laboratory further discovered that MEIG1’s primary function is in germ cells, not in Sertoli cells (9).

The mechanism of MEIG1’s function was further studied in our laboratory. MEIG1 is present in cell bodies of spermatocytes and round spermatids, but it is translocated to the manchette in elongating spermatids (10). The manchette is a transient structure only present in elongating spermatids. The timing of manchette development is very precise (11, 12). Two major functions of the manchette have been proposed: shaping spermatid heads and sorting structural proteins to the centrosome and the developing sperm tail through intra-manchette transport (IMT) (13, 14). These proposed functions are supported by the characteristics of its structural proteins and mutant mouse models. The manchette contains molecular motor proteins and proteins used to build sperm tails (15, 16). Disruption of motor proteins in male germ cells results in spermiogenesis failure associated with a manchette defect (17). Proteins that regulate motor protein function and localization are also present in the manchette, and some of them have been shown to be essential for spermatogenesis (18, 19, 20, 21, 22, 23, 24, 25, 26, 27, 28, 29, 30, 31, 32, 33, 34, 35, 36).

A yeast two-hybrid screen was conducted using full-length mouse MEIG1 as bait to identify binding partners, and PACRG was identified to be its major binding partner (10, 37, 38). *Pacrg* mutant mice showed a similar phenotype as the *Meig1* mutants (39, 40, 41, 42, 43). In germ cells, PACRG protein is not translated until day 28 after birth when germ cells start to elongate, and the translated protein becomes localized in the manchette (10). Using mouse PACRG as bait for a yeast two-hybrid screen, MEIG1 was found to be the major binding partner (10). In elongating spermatids, PACRG determines MEIG1’s localization in the manchette (10).

Sperm-associated antigen 16L (SPAG16L), a long isoform translated from the *Spag16* gene, is a protein localized in the central apparatus of motile cilia (44, 45, 46). SPAG16L is localized in the manchette of elongating spermatids of wild-type mice. However, it is absent in the manchette of the remaining elongating spermatids of MEIG1 or PACRG-deficient mice (10), indicating that SPAG16L is a cargo of MEIG1/PACRG complex. Thus, MEIG1 and PACRG appear to form a complex in the manchette to transport cargo (SPAG16L) to build sperm flagellum.

MEIG1’s structure was resolved by nuclear magnetic resonance (NMR) (47). The shape of MEIG1 resembles a dumbbell, such that associated proteins can bind to either of two opposing concave surfaces or the two convex ends of the dumbbell. Twelve amino acids exposed on the protein surface are believed to mediate interactions between MEIG1 and its binding partners, particularly PACRG (47). We examined the role of the 12 amino acids in MEIG1/PACRG interaction and discovered that the four amino acids located on the same surface, W50, K57, F66, and particularly Y68, mediate interaction between MEIG1 and PACRG (47). We mutated the Y68 amino acid using the CRISPR/cas9 system to study its role *in vivo*. The single amino acid mutant MEIG1 was still present in the cell bodies of spermatocytes and round spermatids, but was no longer present in the manchette of the remaining elongating spermatids. The MEIG1 mutation caused male mice infertility associated with dramatically reduced sperm counts and abnormal sperm morphology, including short tails, vesicles in the flagella, and different thicknesses along the tails. Most importantly, sperm were immotile. SPAG16L was not present in the manchette in elongating spermatids of the mutant mice. The phenotype was similar to the global *Meig1* knockout mice. The study demonstrates that Y68 is a key amino acid for PACRG to recruit MEIG1 to the manchette to form the MEIG1/PACRG complex, which is essential for transporting cargo proteins for sperm formation.

## Results

### Generation of MEIG1 single amino acid (MEIG1^Y68A^) mutant mice

**Figure 1:**
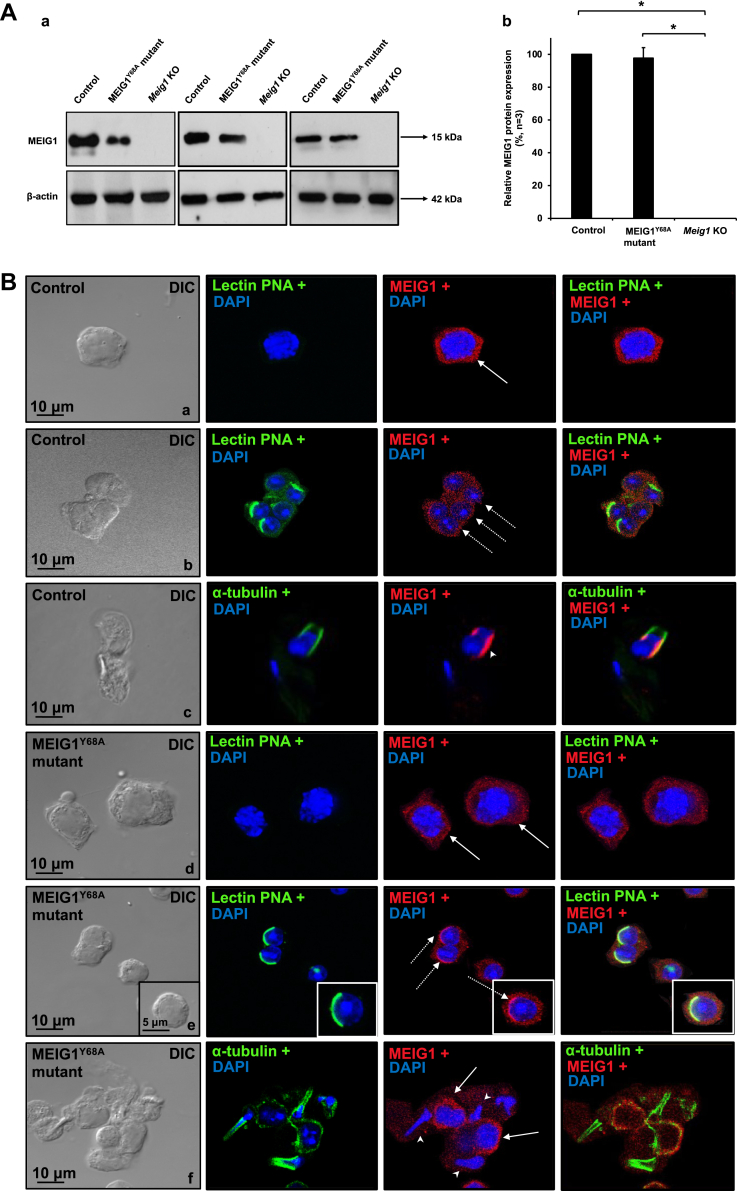
**Generation of MEIG1**^**Y68A**^**mutant mouse model.***A*, examination of testicular MEIG1 expression in the indicated mice. (a) Representative Western blot results to examine testicular MEIG1 protein expression in the control, Y68A homozygous mutant, and global *Meig1* knockout mice. MEIG1 is missing in the global knockout mice, but is still present in the Y68A mutant mice. (b) Quantification of the relative MEIG1 protein expression normalized by β-actin. Error bars represent the standard deviation (n = 3). (∗), *p* < 0.05. *B*, mislocalization of Y68A mutant MEIG1 in round and elongating spermatids. The testicular cells were double stained with MEIG1 antibody (*red*) and the acrosome marker lectin PNA (*green*), or a manchette marker α-tubulin (*green*). Wild-type MEIG1 is localized in cell bodies of spermatocytes (panel a, *arrow*) and round spermatids (panel b, *dashed arrows*), and migrates to the manchette of elongating spermatids (panel c, *arrow head*). Y68A mutant MEIG1 is still present in cell bodies of spermatocytes (panel d, f, *arrows*), but is highly concentrated in the acrosome of round spermatids (panel e, *dashed arrows*); it is not present in the manchette of the remaining elongating spermatids (panel f, *arrow heads*).

We previously discovered that four amino acids, W50, K57, F66, and particularly Y68, mediate interactions between MEIG1 and PACRG *in vitro* (47). To test if these amino acids, particularly Y68, are important for MEIG1’s function *in vivo*, we generated a mouse model replacing Y68 with alanine using the CRISPR/cas9 system (Fig. S1*A*). DNA sequencing of the RT-PCR product revealed that only Y68 amino acid was mutated to A68 in the model (Fig. S1*B*). Western blot analysis using testicular extracts demonstrated that the mutant MEIG1 protein was expressed in the testis (Fig. 1*A*). Localization of the mutant MEIG1 was examined by immunofluorescence staining. As reported previously (10), the wild-type MEIG1 was present in cell bodies of spermatocytes and round spermatids, and it was translocated to the manchette of elongating spermatids (Fig. 1*B*a–c). The Y68A mutant MEIG1 was still present in cell bodies of spermatocytes and round spermatids. However, it was no longer present in the manchette. Interestingly, the mutant MEIG1 appeared to accumulate in the acrosome as an increased MEIG1 signal was observed here (Fig. 1*B*d–f).

### Homozygous MEIG1^Y68A^ mutant mice were infertile associated with reduced sperm number, motility, and increased abnormal sperm in mice

**Figure 2:**
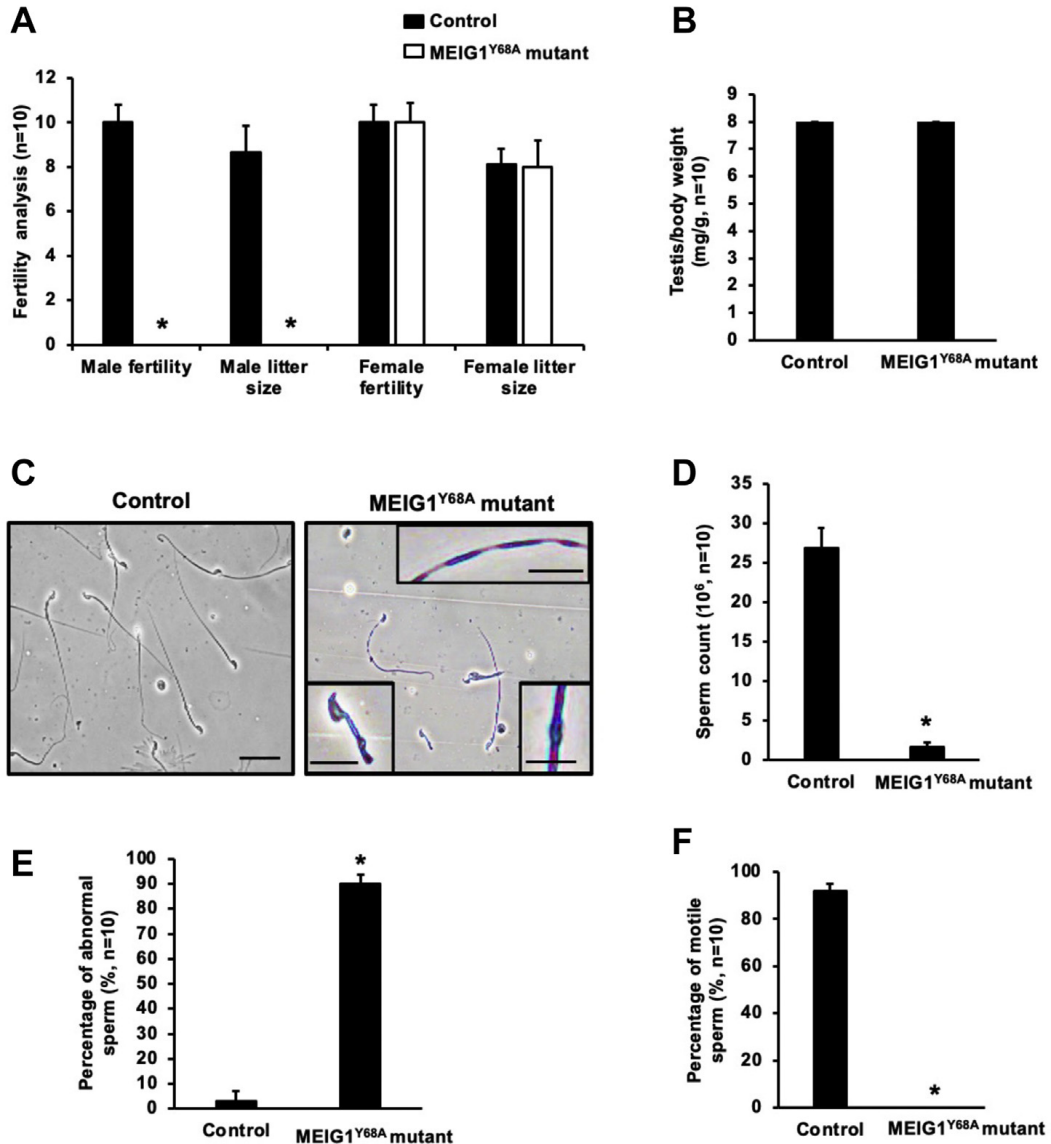
**Y68 single amino acid defect in MEIG1 causes male infertility associated with abnormal sperm morphology, significantly reduced sperm number and motility.***A*, fertility of control and MEIG1^Y68A^ mutant mice. Ten controls and ten MEIG1^Y68A^ mutant mice were examined. Fertility and litter size were recorded for each mating. Notice that all mutant females had normal fertility; however, all mutant males were infertile. *B*, normal testis/body weight of control and Y68A mutant mice. *C*, morphological examination of epididymal sperm from the control (*left*) and mutant (*right*) mice by light microscopy at low magnification. Notice that sperm density of the control mice is higher than those observed in the MEIG1^Y68A^ mice under the same dilution. Sperm in the control mice showed normal morphology, but short tails (*lower, left inset*), vesicles in the flagella (*lower, right inset*), and different thicknesses along the tails (*upper, inset*) were frequently observed in the mutant mice, Bar = 10 μm. *D*, sperm number was significantly reduced in the mutant mice. *E*, percentage of abnormal sperm of control and the mutant mice. *F*, percentage of motile sperm of control and the mutant mice. “n” represents the number of mice analyzed. Data are expressed as Mean ± SD. Statistically significant differences (∗), *p* < 0.05.

Homozygous mutant mice did not show any gross abnormalities. To test fertility of these mutant mice, 2 to 3 month-old wild-type mice and homozygous mutant mice were bred with 2 to 3-month-old wild-type mice for more than 2 months. All the control mice, including wild-type and heterozygous males and homozygous mutant females, showed normal fertility. All homozygous mutant males examined were infertile (Fig. 2*A*). There was no significant difference in testis/body weight between the control and homozygous mutant mice (Fig. 2*B*). Sperm number, morphology, and motility from the control and homozygous mutant mice were examined (Fig. 2, *C*–*F*). The sperm count was dramatically reduced in the mutant mice (Fig. 2, *C* and *D* and Movie S1). Sperm from the control mice showed normal morphology (Fig. 2*C* and Movie S2, left panel). Multiple abnormalities in sperm were observed in the mutant mice, including short tails, vesicles in the flagella, and different thicknesses along the tails (Fig. 2*C* and Movie S1, right panel), and percentage of abnormal sperm was significantly increased in the mutant mice (Fig. 2*E* and Movie S1). More than 70% of sperm from the control group were motile and showed progressive motility (Fig. 2*F* and Movie S2); No sperm were motile in the mutant mice (Fig. 2*F* and Movie S1).

### Abnormal spermiogenesis in the homozygous MEIG1^Y68A^ mutant mice

**Figure 3:**
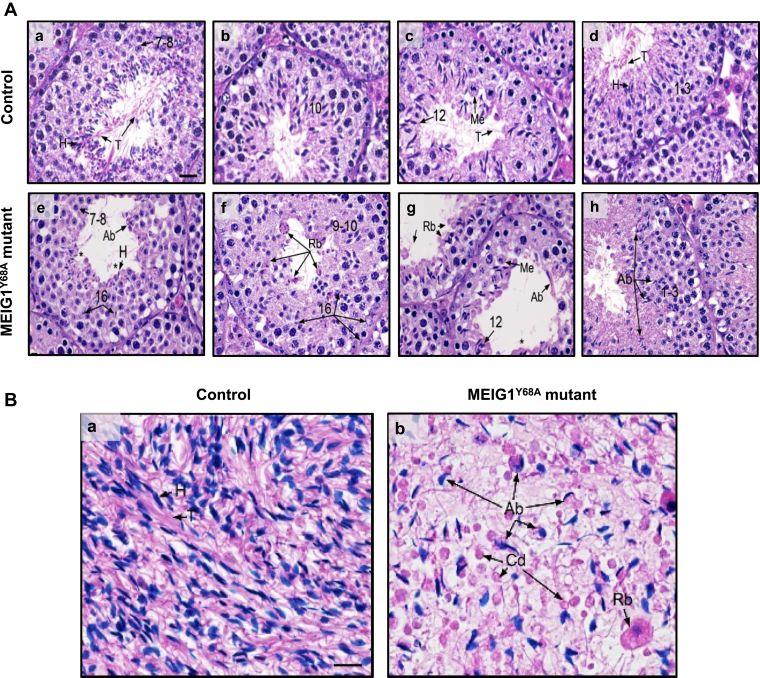
**Spermiogenesis defect in the MEIG1**^**Y68A**^**mutant mice.***A*, testis histology of control (a–d) and homozygous Y68A mutant mice (e–h). (a) Stage VII–VIII showing normal spermatogenesis with step 7 to 8 round spermatids. Step 16 spermatids are aligned by their heads (H) at the lumen, with extensive tails (T). Bar = 25 μm (for all photos). (b) Stage X showing normal step 10 spermatids just beginning to show elongation of the nucleus. (c) Stage XII showing normal meiotic division of spermatocytes (Me) and step 12 elongating spermatids, with their long tails (T) extending into the lumen. (d) Stage I–III showing normal step 1 to 3 round spermatids and elongating spermatids with heads (H) aligned and tails (T) extending into the lumen. (e) Mutant stage VII–VIII showing normal step 7 to 8 round spermatids, but abnormal elongating spermatids (Ab). The abnormal elongating spermatid heads (H) are surrounded by cytoplasm and appear rounded without an extension of the tails (∗). The heads of step 16 spermatids are seen deep within the epithelium, where they have been phagocytized by the Sertoli cells. (f) Mutant stage IX–X showing disorganization of the step 9 to 10 spermatids, with possibly some abnormal shapes. Failure of spermiation is present with rounded cytoplasm of residual bodies (Rb) containing the heads of sperm without tails. Some individual step 16 spermatid heads are also seen having been phagocytized by Sertoli cells. (g) Mutant stage XII showing normal meiotic division (Me) of spermatocytes, but disorganization of the step 12 elongating spermatids. The elongating spermatids are abnormal, lacking tails in many cases (∗) and showing abnormal tails (Ab) with excessive cytoplasm in other cases. (h) Mutant stage I–III showing normal step 1 to 3 round spermatids, but abnormal heads of elongating spermatids (Ab) and few tails extending into the lumen. *B*, the control mouse cauda epididymis showing highly concentrated normal sperm in the lumen with heads (H) and tails (T) aligned (a). The MEIG1^Y68A^ mutant cauda epididymis (b) showing very low concentration of sperm, with high incidence of sperm abnormalities (Ab), including absent tails, excess cytoplasm, and short tails. Round bodies (Rb) in the lumen appear to be larger residual bodies and smaller cytoplasmic droplets (Cd). Few sperm tails are present. Bar = 20 mm (for all photos).

Significantly reduced and nonfunctional sperm suggests impaired spermatogenesis in the mutant mice. To examine the spermatogenesis process in the mutant mice, testes from 4 to 5 month-old control and homozygous mutant mice were collected for HE staining. The control mice showed a normal spermatogenesis process. However, the mutant mice showed impaired spermiogenesis. Elongating spermatids lacking tails or with short tails and deformed heads were frequently observed in the seminiferous tubules of the mutant mice (Fig. 3*A*). Histology of cauda epididymis of the adult control and the mutant mice was also examined. The control mice had highly concentrated normal sperm in the lumen (Fig. 3*B*a). However, the mutant mice had low sperm concentration with multiple abnormalities, as observed in the seminiferous tubules (Fig. 3*B*b).

### Ultrastructural changes in the seminiferous tubules of the MEIG1^Y68A^ mutant mice

**Figure 4:**
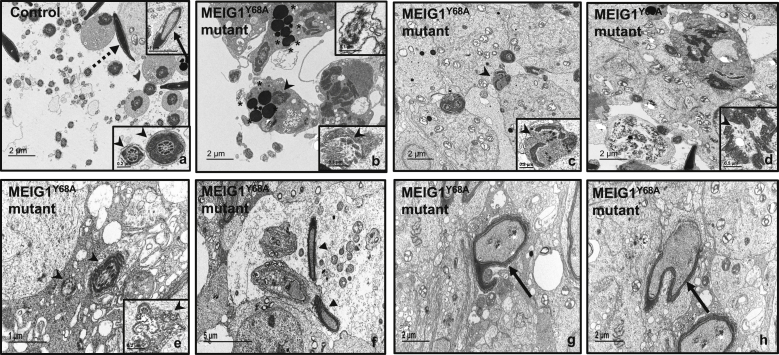
**TEM images of seminiferous tubules of a control mouse and MEIG1**^**Y68A**^**mutant mice.** A number of normally developed sperm were released to the lumen of the seminiferous tubules of a control mouse. Normal chromatin condensation (*dotted arrow*), well-condensed head (*arrow*), and normally developed flagella can be observed. The *arrow heads* point to the middle piece (*right*, *inset*) and the principle piece (*left*, *inset*) in (*a*). (*b*) shows few sperm present in the lumen in the MEIG1^Y68A^ mutant mice. Abnormal “9 + 2” axoneme structure surrounded by abnormally accessory structures (*arrow heads* in *b–e*) were frequently observed. Increased number of the lysosomes were seen in the degrading abnormal flagellum (*stars*, *b*). Multiple elongating spermatids in the seminiferous tubule were wrapped in 1 cell membrane, indicating phagocytized by Sertoli cells (*arrowheads*, *e*). Abnormal condensed head (*triangles*, *f*) and chromatin can be seen in the mutant mice (*arrows*, in *g* and *h*).

To investigate the structural basis for the reduced sperm number and sperm morphology changes, TEM was conducted in testis. In seminiferous tubules of the controls, normal spermiogenesis process was observed, including chromatin condensation, flagella formation, and numbers of elongated spermatids released to the lumen (Fig. 4*a*). However, in the MEIG1^Y68A^ mutant mice, there were few sperm in the seminiferous tubules, and these developed sperm had abnormal flagellar structure, chromatin condensation, and sperm head shapes. The flagella with abnormal ‘‘9 + 2’’ axoneme arrangement were surrounded by many lysosomes, which seemed to degrade the abnormal sperm. They were also concentrated in Sertoli cells, with evidence of Sertoli cell phagocytosis. What’s more, consistent with the phenotype of MEIG1-deficient mice, flagellar components such as microtubules and outer dense fibers could be detected but were not assembled correctly (Fig. 4, *b*–*h*).

### PACRG level and localization were not changed in the MEIG1^Y68A^ mutant mice

**Figure 5:**
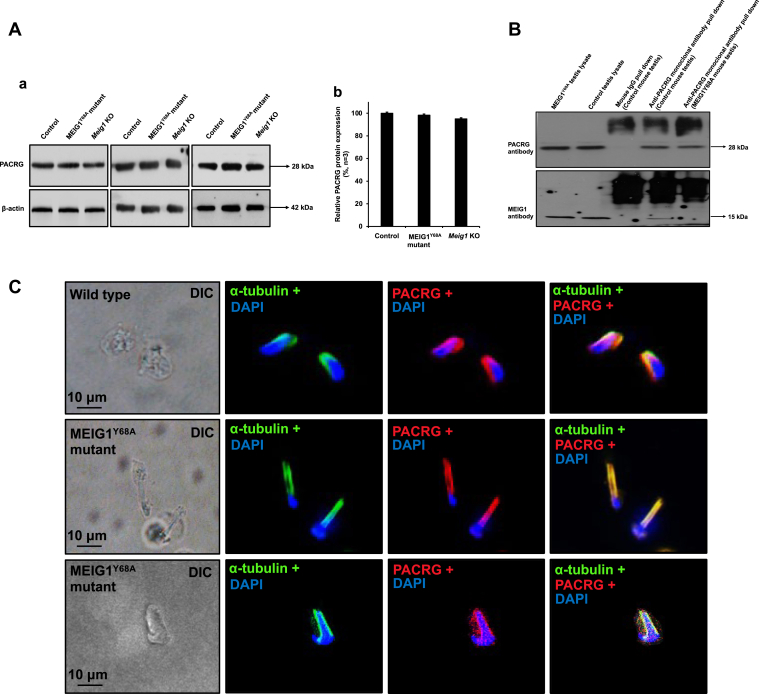
**The localization and expression of PACRG were not changed in the MEIG1**^**Y68A**^**mutant mice.***A*, examination of testicular PACRG expression in the indicated mice by Western blot analysis. (a) Representative Western blot results to examine testicular PACRG protein expression in the control and Y68A homozygous mutant mice; (b) Quantification of the relative PACRG protein expression normalized by β-actin. Error bars represent the standard deviation (n = 3). There was no significant difference in PACRG level between the control and the MEIG1^Y68A^ mutant mice. *B*, PACRG binds to wild-type MEIG1 but not the Y68A mutant MEIG1 in the testis. Co-IP experiment was conducted using testicular lysates from the control and Y68A mutant mice. The PACRG antibody pulled down wild-type MEIG1 but not the mutant MEIG1. *C*, localization of PACRG in germ cells of MEIG1^Y68A^ mutant mice. PACRG is present in the manchette of elongating spermatids in wild-type mice (*upper panel*). In MEIG1^Y68A^ mutant mice, the localization of PACRG was not changed (*middle* and *bottom panels*).

Testicular PACRG level and its localization in elongating spermatids were examined in the MEIG1^Y68A^ mutant mice. There was no difference in PACRG levels between the control and MEIG1 mutant mice (Fig. 5*A*). However, co-IP experiment showed that PACRG does not bind with the Y68A mutant MEIG1 (Fig. 5*B*). We previously discovered that mouse PACRG was present in the manchette of elongating spermatids of wild-type and global *Meig1* knockout mice (10). In the MEIG1^Y68A^ mutant mice, like in the control mice (Fig. 5*C*, top panels), PACRG was still present in the manchette (Fig. 5*C*, bottom two panels), even though the mutant MEIG1 was not present in the manchette anymore (Fig. 1*B*f), supporting our previous conclusion that PACRG is an “upstream protein” of MEIG1.

### SPAG16L, the cargo of MEIG1/PACRG complex, is absent from the manchette of elongating spermatids of the MEIG1^Y68A^ mutant mice

**Figure 6:**
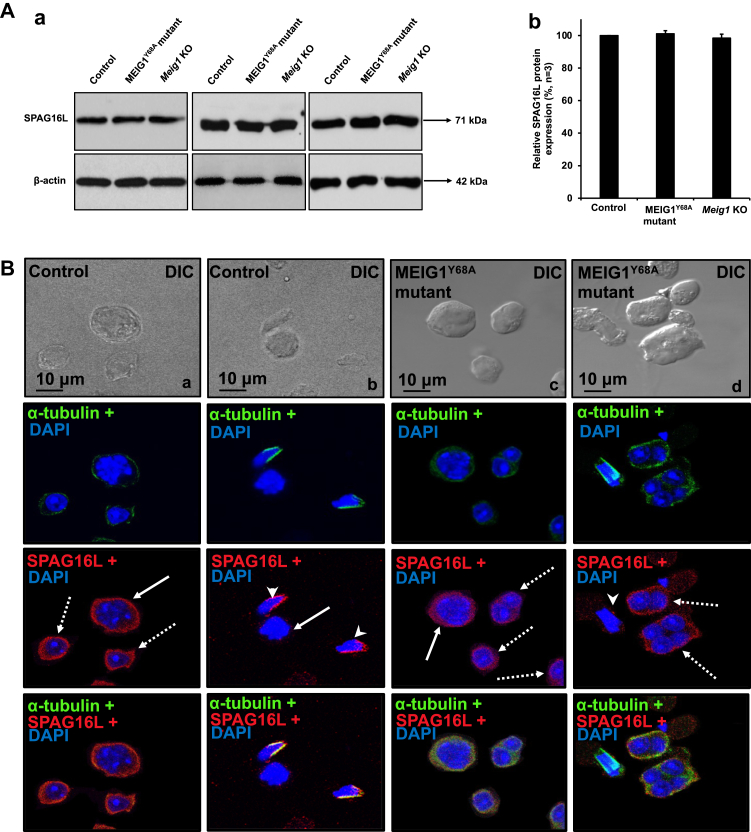
**MEIG1**^**Y68A**^**mutation changes localization of SPAG16L in elongating spermatids.***A*, examination of testicular SPAG16L expression in the indicated mice by Western blot analysis. (a) Representative Western blot results to examine testicular SPAG16L protein expression in the control and Y68A homozygous mutant mice; (b) Quantification of the relative SPAG16L protein expression normalized by β-actin. Error bars represent the standard deviation (n = 3). There was no significant difference in SPAG16L level between the control and the MEIG1^Y68A^ mutant mice. *B*, localization of SPAG16L in germ cells of wild-type and MEIG1^Y68A^ mutant mice. In control mice, SPAG16L staining (*red*) was observed in the cytoplasm of spermatocytes (panel a, b, *arrows*) and round spermatids (panel a, *dotted arrows*), and the manchette of elongating spermatids (panel b, *arrow heads*), as evaluated by double staining with an anti-α-tubulin antibody (*green*). In MEIG1^Y68A^ mutant mice, SPAG16L is still present in cytoplasm of spermatocytes (panel c, *arrow*) and round spermatids (panel c, *dotted arrows*); however, it is not present in the manchette of elongating spermatids (panel d, *arrow head*).

SPAG16L is present in the cytoplasm of spermatocytes and round spermatids and is recruited to the manchette by MEIG1/PACRG complex (10). We therefore examined testicular SPAG16L expression levels and localization in the MEIG1^Y68A^ mutant mice. The testicular SPAG16L expression level was not changed as revealed by Western blot analysis (Fig. 6*A*). In spermatocytes and round spermatids, SPAG16L was still localized in the cytoplasm of the mutant mice, which is consistent with the localization as seen in the control mice (Fig. 6*B*a–d). However, SPAG16L was no longer present in the manchette in elongating spermatids of the mutant mice (Fig. 6*B*d). The results further support our conclusion that the manchette localization of SPAG16L is dependent on MEIG1 (10).

## Discussion

Our lab previously resolved MEIG1’s structure by NMR. Twelve amino acids exposed on the protein surface are believed to mediate interactions between MEIG1 and its binding partners (47). We further examined the role of the 12 amino acids on MEIG1’s surface in MEIG1/PACRG interaction and discovered that the four amino acids located on the same surface, W50, K57, F66, and especially Y68, are involved in interaction with PACRG (47). In this study, we demonstrated that Y68 is essential for MEIG1’s function *in vivo*. We discovered that the male reproductive phenotype of MEIG1 Y68A mutants is consistent with what was observed in global *Meig1* KO mice. Homozygous mutant mice did not show any gross abnormalities. However, all homozygous mutant mice were infertile associated with multiple defects in spermiogenesis.

Although the Y68A mutant MEIG1 was still in cell bodies of spermatocytes and round spermatids, it was no longer present in the manchette. This is consistent with our previous conclusion that MEIG1 localization in the manchette is dependent on PACRG (10). During the first wave of spermatogenesis, PACRG is expressed at postnatal day 28, when germ cells start to condense and the manchette is formed (10). PACRG recruits MEIG1 through binding to the Y68 amino acid to form a MEIG1/PACRG complex in the manchette. When Y68 on the MEIG1 is mutated, PACRG is unable to bind to MEIG1, and therefore the mutant MEIG1 is no longer present in the manchette. Interestingly, even though MEIG1 is not concentrated in the acrosome region of round spermatids in the wild-type mice, the mutant MEIG1 seems to accumulate there. It has been proposed that acrosome-derived vesicles contribute to the manchette components (14, 15). Accumulation of the mutant MEIG1 in the acrosome suggests that partial wild-type MEIG1 is recruited to the manchette through the acrosome–manchette pathway. When the MEIG1 is mutated, this pathway is disrupted, and the mutant MEIG1 stays in the acrosome.

It is not surprising that PACRG was still present in the manchette of remaining elongating spermatids when MEIG1 was mutated. This result further confirms that PACRG is an “upstream protein” of MEIG1 (10). We previously discovered that SPAG16L, a component of the central apparatus of motile cilia, is a cargo protein of MEIG1/PACRG complex. SPAG16L is localized in the manchette of elongating spermatids of wild-type mice, and the localization is MEIG1 or PACRG dependent (10). Similar localization was observed in the present model: when MEIG1 was mutated, SPAG16L was still present in the cytoplasm of spermatocytes and round spermatids, but not in the manchette. This again confirms that SPAG16L localization in the manchette depends on the correct localization of MEIG1 in the manchette. These results highlight the function of the MEIG1/PACRG complex in the manchette for transporting cargo proteins for sperm flagella formation.

**Figure 7:**
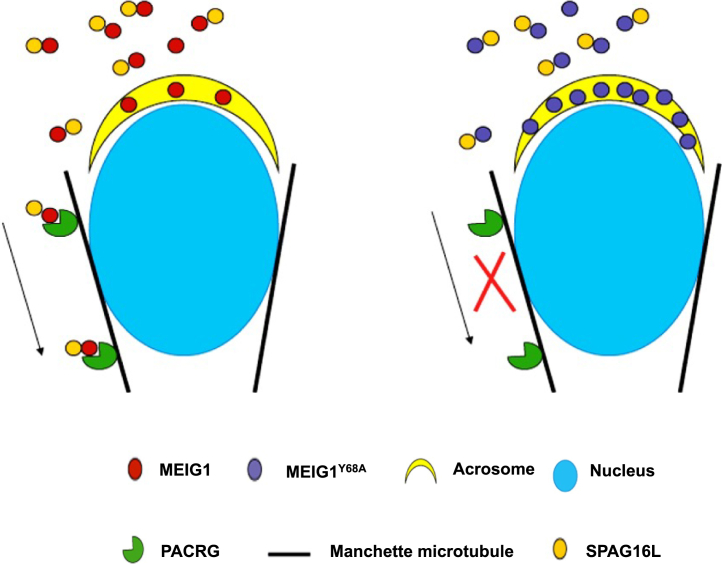
**Working model of MEIG1/PACRG complex in transporting cargo in the manchette.** MEIG1 is recruited to the manchette by PACRG. SPAG16L, a sperm structure protein localized in the central apparatus of axoneme is a cargo of MEIG1/PACRG complex. Disruption of MEIG1/PACRG complex, such as mutation of Y68 on the MEIG1 protein shuts down the transport system.

According to the above results, we propose a working model of MEIG1/PACRG complex in transporting cargo in the manchette for normal spermiogenesis and sperm formation. PACRG recruits MEIG1 to the manchette through binding with specific amino acids. The MEIG1/PACRG complex then associates with cargo proteins including SPAG16L. Disruption of interaction between PACRG and MEIG1, for example, mutating the key amino acids that mediate MEIG1/PACRG interaction, shuts down the transport system in the manchette, which will ultimately result in failure of sperm flagella formation and impaired spermiogenesis (Fig. 7).

In this study, we only mutated Y68. Even though the homozygous males were completely infertile, the sperm number was higher than that in the global *Meig1* knockout mice (2), suggesting that other amino acids also play a role *in vivo*. Some amino acids, including W50, might function through forming the MEIG1/PACRG complex for cargo transporting in the manchette. However, it remains unknown if other amino acids that do not mediate MEIG1/PACRG interaction are also essential for spermatogenesis.

It has been shown that acrosome-derived vesicles contribute to the manchette components transport (14). Even though the manchette backbone is still present in the mutant mice, only few sperm are formed in the mutant mice. We expect that the manchette transport of most MEIG1-dependent acrosome-derived vesicles was affected. However, some vesicles not dependent on MEIG1 might still be transported in the manchette. Further studies need to be conducted to investigate MEIG1-dependent and independent manchette transport in the future.

The interaction between MEIG1 and PACRG is conserved in humans. The structure of human MEIG1/PACRG complex has been recently solved, including identification of the key amino acids on PACRG protein that mediate the MEIG1/PACRG interaction (48). Variation in the *PACRG* promoter has been shown to be a risk factor associated with azoospermia (49). A recent exome sequencing study revealed that PACRG is a novel candidate gene for severe sperm motility disorders (50). However, no human MEIG1 mutations have been reported to be associated with infertility. Though the MEIG1/PACRG complex has not been implicated in male infertility, it is known that asthenozoospermia, or low sperm motility, is found in 80%, to varying degrees, of infertile men (51). Asthenozoospermia is caused, to a large extent, by morphological or functional defects in the sperm flagella. Almost 40% of genes that cause isolated male infertility are related to flagella function, with 18 monogenic disease genes having been identified to date (52). These genes that affect the development of the sperm flagella, such as the MEIG1/PACRG complex, would likely have an impact on human fertility.

In males, hormone-based contraceptives can have numerous adverse side effects, necessitating the identification and validation of new molecular targets for male contraceptive drugs. One attractive strategy is to target the unique biologic processes that are required for spermatogenesis. Compounds that target these processes would be expected to have improved specificity, efficacy, and onset of action with fewer side effects compared with hormonal contraceptives. Our data provide compelling genetic validation for the MEIG1/PACRG complex as a novel target for nonhormonal contraceptive drugs. The fact that this essential interaction depends on the single amino acid Y68 in the interaction interface strongly suggests that the interaction could be disrupted by small synthetic molecules.

## Experimental procedures

### Ethics statement

Animal research were performed in accordance with Federal and local regulations regarding the use of nonprimate vertebrates in scientific research and approved by Wayne State University Institutional Animal Care and Use Program Advisory Committee (Protocol number: IACUC-18-02-0534).

### Generation of MEIG1^Y68A^ mutant mouse model

To introduce the Y68A mutation into *Meig1*, single guide (sg) RNA 5′- CTT CTA CTA CAA CAA AGA GA was selected because of its high score with minimum off-target effects. This sequence was designed to cleave in exon 4 of *Meig1* with adjoining GGG, a protospacer adjacent motif (PAM) and encompassing the site of Y68A knockin. An ssDNA donor with sequences encoding *Meig1* with the TAC > **GC**C: Y68A mutation and a silent mutation AGG > AG**A** together with 5′ and 3′ homology arms was used as a template for homology-directed repair to knock-in the mutation. The donor sequence to be used is: 5′- G∗G∗ A∗AA AGG TCA GTA GAC GTA AAC CTT CAC TTT GTG GAC CTC CTT GTC CTC GCA CTC **T**CT CTC TTT GTT GTA G**GC** GAA GAA AGT ATT GTC CCT CCG CTG AAG TTT CTT CAC GTA CCC TGT CTC TGG CCA TCG GTC GAC CTG CA∗C∗ C∗G, where **GC** is the TAC > **GC**C knock-in mutation and T is the AGG > AG**T** silent mutation, which was introduced to eliminate the PAM site to prevent recleavage of knockin alleles by the CRISPR RNP. ∗ denotes phosphorothioate linkage to prevent exonuclease-mediated degradation. The donor ssDNA and sgRNA were ordered from IDT.

The injection mix, which consists of 30 ng/μl of Cas9 protein, 50 ng/μl of sgRNA, and 50 ng/μl ssDNA donor, was microinjected into the pronucleus of C57BL6 one-cell embryos and then transferred to pseudopregnant females for subsequent development. Live born pups were initially screened by PCR using primers: Meig1 I3F 5′- GTC AGA CGG TGA AAC GTG ACG and Y68Ar 5′- CAC TCT CTC TCT TTG TTG TAG GC to amplify a fragment of 158 bp specific to the Y68A knockin allele. Potential founders were further screened by PCR using primers: Meig1 I3F 5′- GTC AGA CGG TGA AAC GTG ACG and Meig1 3UTRR 5′- GCT GCC TGG AGC ACA AAT GTG to amplify a fragment of 526 bp encompassing the TAC > **GC**C: Y68A knockin mutation. The PCR products were sequenced to confirm the identity of the founders.

### Western blots analysis and coimmunoprecipitation

For Western blot analysis, freshly excised or frozen whole testes were collected from 4 to 5-month-old mice and homogenized in buffer supplemented with 50 mM Tris-HCl, pH 8.0, 170 mM NaCl, 1% NP40 (Sigma-Aldrich, 127087-87-0), 5 mM EDTA, 1 mM DTT and protease inhibitors (Complete mini; Roche diagnostics GmbH, 11836170001). Protein was quantified using Bio-Rad DCTM protein assay kit (Bio-Rad, 5000121) and normalized to equal concentration in SDS loading buffer. Protein electrophoresis was performed, and the protein was transferred to PVDF membranes (Millipore/EMD) using the wet transfer system. Then, PVDF membranes were incubated with the following antibodies: MEIG1 (1:10,000 dilution); ACTB/β-actin (1:2000; Cell Signaling Technology, 4967S); SPAG16L (1:2000 dilution); PACRG (1:2000 dilution). Finally, Western blot was developed using Super Signal West Pico chemiluminescent substrate.

For coimmunoprecipitation assay, freshly excised whole testes from 4 to 5-month-old control or MEIG1^Y68A^ mutant mice were used following the same procedure as described previously (2) except that a monoclonal anti-PACRG antibody was used for the pull-down process (10).

### Sperm counts and morphology analysis

After euthanasia, the cauda epididymis was immediately removed from each mouse and placed in 1 ml of warm phosphate-buffered saline (PBS) solution (Thermo Fisher Scientific, 10010023) at 37 °C. Two openings were cut in each epididymis to allow sperm to swim out. Sperm were collected and fixed with 4% formaldehyde for 10 min at room temperature (Sigma-Aldrich, 252549). Sperm number was counted using a hemocytometer chamber under a light microscope. To analyze sperm morphology, images were taken with a ProgRes C14 camera (Jenoptik Laser) under a BX51 Olympus microscope.

### Tissue histology

For histology analysis, testis and cauda epididymis of adult mice were fixed in 4% formaldehyde prior to embedding in paraffin wax. The tissues were sectioned at 5 μm and stained with hematoxylin and eosin (HE, Abcam, ab245880). Images were taken as described above.

### Isolating spermatogenic cells and immunofluorescence staining

Testes from 4 to 5-month-old mice were dissected and placed in cold 1× PBS to wash off any contaminates. The tunica albuginea was discarded and the seminiferous tubules were released in 5 ml DMEM containing 0.5 mg/ml collagenase IV (Sigma-Aldrich, c1889-50mg) and 1.0 mg/ml DNase I (Sigma-Aldrich, dn25-1g). Afterward the seminiferous tubules were incubated, with constant shaking, at 32 °C for 30 min. After the testicular cells were dissociated, they were centrifuged at 160*g* for 5 min. Supernatant was poured out and washed three times with 5 ml cold 1× PBS each time. The dispersed mixed testicular cells were fixed in 4% paraformaldehyde/PBS (containing 4% sucrose), shaken for 15 min at room temperature, then washed three times with cold 1× PBS. Cells were resuspended in 1 ml cold 1× PBS and plated on SuperFrost/Plus microscope slides (Thermo Fisher Scientific, 22-037-246). After air-drying, the cells were permeabilized with 0.1% Triton X-100 at 37 °C for 5 min on a wet box and then washed with 1× PBS three times. Cells were blocked with 10% goat serum in PBS at 37 °C for 30 min. Then the following primary antibodies were incubated at 4 °C overnight: anti-MEIG1 (1:1000, dilution), SPAG16L (1:200 dilution); PACRG (1:200 dilution); Peanut-lectin (2 μg/ml, Invitrogen, L21409). The primary antibodies were removed and washed with 1× PBS three times. The slides were incubated with Cy3-conjugated anti-rabbit IgG secondary antibody (1:5000; Jackson ImmunoResearch Laboratories, 111-165-003) for 1 h at room temperature. The secondary antibodies were discarded and washed three times with 1× PBS. Finally, the antibodies were mounted with VectaMount containing DAPI (Vector Laboratories, h-1800) and sealed with a coverslip. Images were taken at the Wayne State University Microscopy Core facility by using a confocal microscopy.

### Transmission electron microscopy

Adult testes were fixed with 3% glutaraldehyde in 0.1 M sodium cacodylate (pH of 7.4) at 4 °C overnight and processed for electron microscopy analysis. Images were taken by using a Jeol JEM-1230 transmission electron microscope.

### Statistical analysis

All data were presented as mean ± SEM. Graphs were created using Microsoft Excel. The statistical significance of the difference between the mean values for the different genotypes was examined using Student's *t* test. *p* > 0.05 was considered as not significant and by convention ∗*p* < 0.05.

## Data availability

The location of the data described in the manuscript is indicated and all data are contained within the manuscript.

## Supporting information

This article contains supporting information.

## Conflict of interest

The authors declare that they have no conflicts of interest with the contents of this article.

## Supporting information

Supplemental Movie S1 Representative movies from a MEIG1Y68A mutant mouse. Notice that there are fewer sperm compared with the control mice in the same dilution, and almost all sperm are immotile.

Supplemental Movie S2 Representative movie from a control mouse. Note that most sperm are motile and display vigorous flagellar activity and progressive long-track forward movement.

Supplemental Figure S1 Y68 was mutated to A68 in MEIG1 by CRISPR/cas9 system. A. Schematic diagram of generation of MEIG1 Y68A knock-in mouse strain. B. DNA sequencing of an RT-PCR product amplified from a testicular cDNA from a homozygous Y68A mutant mouse. Only Y68 (TAC) was mutated to A68 (GCC) in the *Meig1* cDNA.
