# Supplementary material for: A single amino acid mutation in the mouse MEIG1 protein disrupts a cargo transport system necessary for sperm formation
Source: J Biol Chem. 2021 Oct 19;297(5):101312. doi: 10.1016/j.jbc.2021.101312 (PMC8592874; doi:10.1016/j.jbc.2021.101312)

Supplemental Figure 1

A

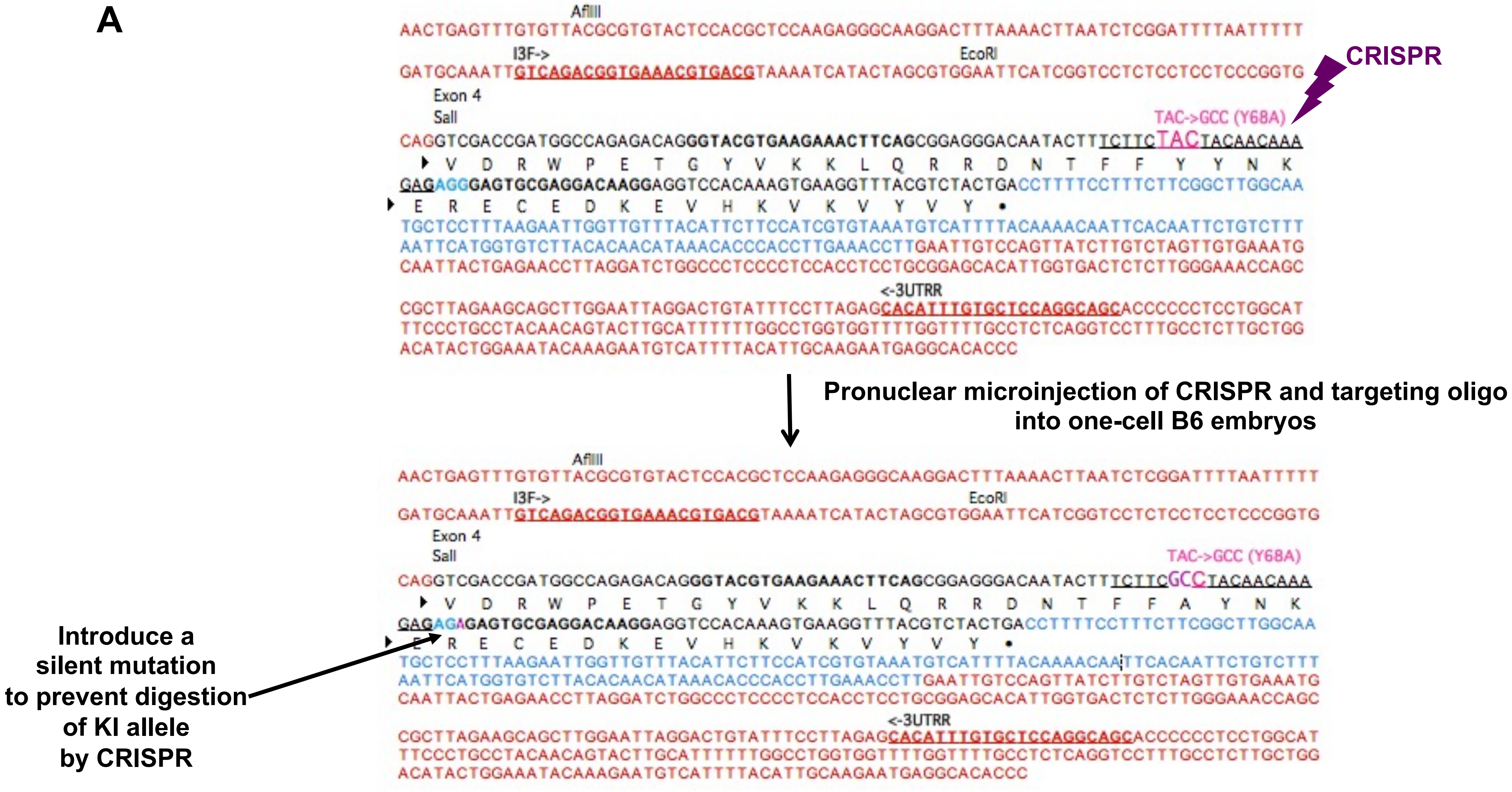

Supplemental Figure 1

B

Wild type

MEIG1^{Y68A} mutant

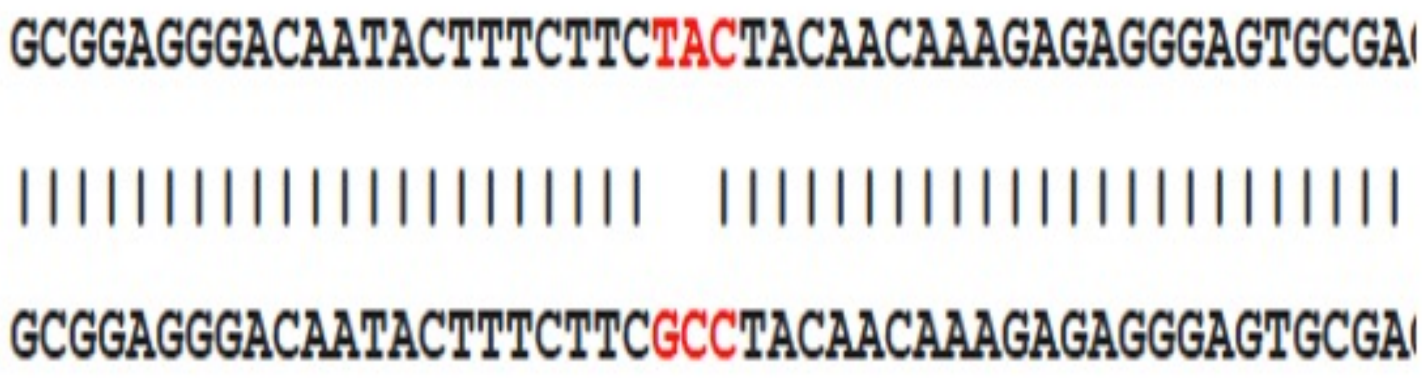

Supplement: Supplemental Figure S1 — Y68 was mutated to A68 in MEIG1 by CRISPR/cas9 system. A. Schematic diagram of generation of MEIG1 Y68A knock-in mouse strain. B. DNA sequencing of an RT-PCR product amplified from a testicular cDNA from a homozygous Y68A mutant mouse. Only Y68 (TAC) was mutated to A68 (GCC) in the Meig1 cDNA. [file mmc3.pdf]
